# Burden of cardiovascular diseases in the Eastern Mediterranean Region, 1990–2015: findings from the Global Burden of Disease 2015 study

**DOI:** 10.1007/s00038-017-1012-3

**Published:** 2017-08-03

**Authors:** GBD 2015 Eastern Mediterranean Region Cardiovascular Disease Collaborators, Arash Tehrani-Banihashemi, Maziar Moradi-Lakeh, Charbel El Bcheraoui, Raghid Charara, Ibrahim Khalil, Ashkan Afshin, Michael Collison, Farah Daoud, Kristopher J. Krohn, Adrienne Chew, Leslie Cornaby, Kyle J. Foreman, Joseph Frostad, Nicholas J. Kassebaum, Laura Kemmer, Michael Kutz, Patrick Liu, Mojde Mirarefin, Grant Nguyen, Haidong Wang, Ben Zipkin, Amanuel Alemu Abajobir, Marian Abouzeid, Niveen M. E. Abu-Rmeileh, Aliasghar Ahmad Kiadaliri, Muktar Beshir Ahmed, Baran Aksut, Khurshid Alam, Deena Alasfoor, Raghib Ali, Reza Alizadeh-Navaei, Rajaa Al-Raddadi, Ubai Alsharif, Khalid A. Altirkawi, Nelson Alvis-Guzman, Nahla Anber, Palwasha Anwari, Johan Ärnlöv, Solomon Weldegebreal Asgedom, Tesfay Mehari Atey, Ashish Awasthi, Till Bärnighausen, Umar Bacha, Aleksandra Barac, Suzanne L. Barker-Collo, Neeraj Bedi, Derrick A. Bennett, Derbew Fikadu Berhe, Sibhatu Biadgilign, Zahid A. Butt, Jonathan R. Carapetis, Ruben Estanislao Castro, Abdulaal A. Chitheer, Kairat Davletov, Samath D. Dharmaratne, Shirin Djalalinia, Huyen Phuc Do, Manisha Dubey, Hedyeh Ebrahimi, Babak Eshrati, Alireza Esteghamati, Maryam S Farvid, Seyed-Mohammad Fereshtehnejad, Florian Fischer, Solomon Weldemariam Gebrehiwot, Tsegaye Tewelde Gebrehiwot, Richard F. Gillum, Philimon N. Gona, Rajeev Gupta, Nima Hafezi-Nejad, Randah Ribhi Hamadeh, Samer Hamidi, Mohamed Hsairi, Sun Ha Jee, Jost B. Jonas, Chante Karimkhani, Amir Kasaeian, Yousef Saleh Khader, Ejaz Ahmad Khan, Daniel Kim, Dharmesh Kumar Lal, Heidi J. Larson, Asma Abdul Latif, Shai Linn, Paulo A. Lotufo, Raimundas Lunevicius, Hassan Magdy Abd El Razek, Azeem Majeed, Reza Malekzadeh, Deborah Carvalho Malta, Toni Meier, Peter Memiah, Ziad A. Memish, Walter Mendoza, George A. Mensah, Atte Meretoja, Ted R. Miller, Erkin M. Mirrakhimov, Shafiu Mohammed, Quyen Le Nguyen, Vuong Minh Nong, Jonathan Pearson-Stuttard, Farhad Pishgar, Farshad Pourmalek, Mostafa Qorbani, Amir Radfar, Anwar Rafay, Vafa Rahimi-Movaghar, Rajesh Kumar Rai, Saleem M. Rana, David Laith Rawaf, Salman Rawaf, Andre M. N. Renzaho, Satar Rezaei, Kedir Teji Roba, Gholamreza Roshandel, Mahdi Safdarian, Sare Safi, Saeid Safiri, Mohammad Ali Sahraian, Payman Salamati, Abdallah M. Samy, Milena M. Santric Milicevic, Benn Sartorius, Sadaf G. Sepanlou, Masood Ali Shaikh, Diego Augusto Santos Silva, Jasvinder A. Singh, Badr H. A. Sobaih, Konstantinos Stroumpoulis, Rizwan Suliankatchi Abdulkader, Cassandra E. I. Szoeke, Mohamad-Hani Temsah, Bach Xuan Tran, Kingsley Nnanna Ukwaja, Olalekan A. Uthman, Tommi Vasankari, Vasiliy Victorovich Vlassov, Stein Emil Vollset, Tolassa Wakayo, Robert G. Weintraub, Priscilla R. Wessly, Tissa Wijeratne, Charles D. A. Wolfe, Abdulhalik Workicho, Mohsen Yaghoubi, Yuichiro Yano, Mehdi Yaseri, Naohiro Yonemoto, Mustafa Z. Younis, Chuanhua Yu, Maysaa El Sayed Zaki, Aisha O. Jumaan, Theo Vos, Gregory A. Roth, Simon I. Hay, Mohsen Naghavi, Christopher J. L. Murray, Ali H. Mokdad

**Affiliations:** Institute for Health Metrics and Evaluation, University of Washington, Seattle, WA USA

**Keywords:** Cardiovascular disease, Burden of disease, Eastern Mediterranean Region

## Abstract

**Objectives:**

To report the burden of cardiovascular diseases (CVD) in the Eastern Mediterranean Region (EMR) during 1990–2015.

**Methods:**

We used the 2015 Global Burden of Disease study for estimates of mortality and disability-adjusted life years (DALYs) of different CVD in 22 countries of EMR.

**Results:**

A total of 1.4 million CVD deaths (95% UI: 1.3–1.5) occurred in 2015 in the EMR, with the highest number of deaths in Pakistan (465,116) and the lowest number of deaths in Qatar (723). The age-standardized DALY rate per 100,000 decreased from 10,080 in 1990 to 8606 in 2015 (14.6% decrease). Afghanistan had the highest age-standardized DALY rate of CVD in both 1990 and 2015. Kuwait and Qatar had the lowest age-standardized DALY rates of CVD in 1990 and 2015, respectively. High blood pressure, high total cholesterol, and high body mass index were the leading risk factors for CVD.

**Conclusions:**

The age-standardized DALY rates in the EMR are considerably higher than the global average. These findings call for a comprehensive approach to prevent and control the burden of CVD in the region.

**Electronic supplementary material:**

The online version of this article (doi:10.1007/s00038-017-1012-3) contains supplementary material, which is available to authorized users.

## Introduction

The Global Burden of Disease (GBD) study documented that cardiovascular diseases (CVD) have been the leading cause of global mortality since 1980 (Institute for Health Metrics and Evaluation 2017; Mortality and Causes of Death 2016). CVD accounted for nearly one-third of all deaths worldwide in 2015. Meanwhile, the principal components of CVD, namely stroke and ischemic heart disease, accounted for 85.1% (95% uncertainty interval (UI): 84.7–85.5) of all deaths in the CVD category in 2015 (Mortality and Causes of Death 2016).

Although the age-standardized mortality rates of CVD have fallen by 27.3% in the last 25 years, the absolute number of deaths due to CVD increased globally by 42.4% between 1990 and 2015 (2017). Most CVD deaths occur in low- and middle-income countries (Mensah et al. 2015). The decline in age-standardized rates is mainly due to preventive interventions and better access to quality treatment for acute cardiovascular conditions such as myocardial infarction and stroke (Smith 2011). CVD also impose a high economic burden on health systems and society. For instance, CVD personal spending in the United States was estimated to be 231.1 billion USD in 2013 and was the largest disease category of personal health care spending (Dieleman et al. 2016).

The Eastern Mediterranean Region (EMR) comprises 22 countries with a population of nearly 580 million people, with a diverse range in per capita gross national product (maximum 83,990 USD for Qatar, minimum 610 USD for Afghanistan) (World Development Indicators database 2017). To the best of our knowledge, there is no comprehensive report on the burden and mortality of CVD in the EMR.

This study aimed to report findings on cardiovascular diseases between 1990 and 2015, from the Global Burden of Diseases, Injuries and Risk Factors Study (GBD 2015) in the 22 countries of the EMR. This would be help us better understand the burden of CVD and interventions needed to control these diseases.

## Methods

GBD 2015 covers 195 countries, 21 regions, and seven super-regions from 1990 to 2015 for 315 diseases and injuries, 2619 unique sequelae, and 79 risk factors by age and sex. Detailed descriptions of the general methodological approach of GBD 2015 and specific methodology used for CVD have been provided elsewhere (GBD 2015 DALYs and Collaborators 2016; GBD 2015 Disease and Injury Prevalence Collaborators 2016; GBD 2015 Mortality and Causes of Death Collaborators 2016).

We evaluated the burden of CVD in the Eastern Mediterranean Region (EMR), which contains 22 countries: Afghanistan, Bahrain, Djibouti, Egypt, Iran, Iraq, Jordan, Kuwait, Lebanon, Libya, Morocco, Oman, Pakistan, Palestine, Qatar, Saudi Arabia, Somalia, Sudan, Syria, Tunisia, the United Arab Emirates (UAE), and Yemen.

The category of CVD includes the ten most common global causes of CVD-related death: rheumatic heart disease, ischemic heart disease, cerebrovascular disease (ischemic stroke and hemorrhagic stroke), hypertensive heart disease, cardiomyopathy and myocarditis, atrial fibrillation and flutter, aortic aneurysm, peripheral vascular disease, endocarditis, and “other cardiovascular and circulatory diseases.” Electronic supplementary table S1 shows the International Classification of Diseases (ICD-10) codes for each of the cardiovascular causes.

To estimate the number of deaths due to CVD, we estimated all-cause mortality envelopes (total number of deaths) for each country-year during 1990–2015; we used all accessible data such as vital registration systems, sample registration data, and household recall of deaths. These sources were used as inputs for cause of death models. We used cause of death ensemble modeling (CODEm) to estimate the number of deaths for each CVD by age, sex, country, and year. The number of deaths for each cause and life tables for all-cause mortality were used to calculate years of life lost (YLLs) (GBD 2015 Morality and Causes of Death Collaborators 2016; Roth et al. 2015a, b).

We updated our previous systematic reviews for the GBD study separately for each of the non-fatal sequelae of CVD. Data on epidemiologic measures (incidence, prevalence, and case fatality) were extracted from 170 data sources. List of all sources (by cause and location) are available at the Institute for Health Metrics and Evaluation’s website (IHME 2016).

Bayesian meta-regression analysis through DisMod-MR 2.1 was used for disease modeling. Model-based prevalence estimates, in combination with disability weights, were used to calculate cause-specific years lived with disability (YLDs) for each age, sex, location, and year. Disability-adjusted life years (DALYs) were calculated through summation of YLLs and YLDs (DALYs and Collaborators 2016; Disease et al. 2016).

We report 95% uncertainty intervals (UI) for each estimate, including rates, numbers of deaths, and DALYs. We estimated UIs by taking 1000 samples from the posterior distribution of each quantity and using the 25th- and 975th-ordered draws of the uncertainty distribution.

## Results

### Mortality

**Fig. 1:**
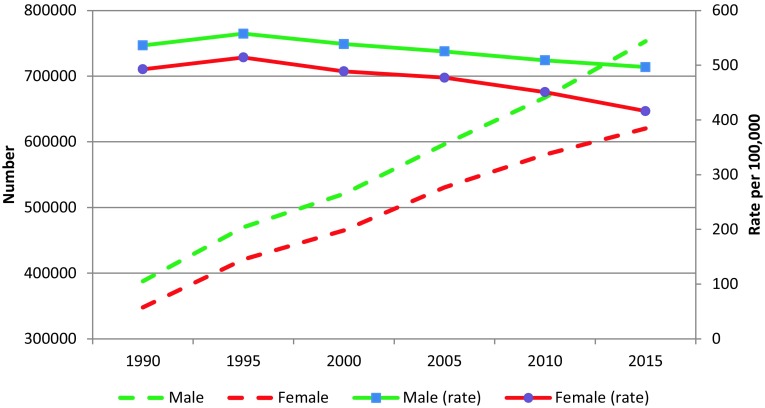
Trend of number of deaths and age-standardized mortality rate from cardiovascular diseases in males and females, Global Burden of Disease study, Eastern Mediterranean Region, 1990–2015

The CVD death rate per 100,000 population in the EMR decreased from 515.1 (95% UI: 491.7–541.5) in 1990 to 456.5 (95% UI: 431.5–484.2) in 2015 (Table 1). A total of 1,373,329 CVD deaths (95% UI: 1,290,959–1,465,047) occurred in 2015 in the EMR, 54.8% of which were among males. These deaths accounted for 34.1% (95% UI: 33.1–35.1) of all deaths in the region in 2015, compared to 30.2% (95% UI: 29.5–30.9) of all deaths in 1990. The number of men dying from CVD was consistently higher than the number of women during 1990–2015 (Fig. 1).

**Table 1:** Total number of deaths and age-standardized mortality rates for cardiovascular diseases in 1990 and 2015, and percentage change, Global Burden of Disease study, Eastern Mediterranean Region, 1990–2015

Cause	Number of deaths					Age-standardized death rate per 100,000				
	1990		2015		% Change	1990		2105		% Change
	Number	95% UI	Number	95% UI		Rate	95% UI	Rate	95% UI	
Cardiovascular diseases	735,689	700,875–773,593	1,373,329	1,290,959–1,465,047	86.7	515.1	491.7–541.5	456.5	431.5–484.2	−11.4
Rheumatic heart disease	18,350	16,029–21,037	27,046	22,945–31,078	47.4	9.1	7.8–10.7	6.8	5.7–7.8	−25.5
Ischemic heart disease	403,355	379,184–425,913	802,078	750,839–859,266	98.9	294.0	276.9–310.3	269.1	252.5–286.9	−8.5
Ischemic stroke	92,230	79,786–106,780	174,760	158,325–190,197	89.5	75.5	65.5–87.8	65.6	59.7–71.2	−13.1
Hemorrhagic stroke	117,813	105,731–133,751	200,113	182,283–230,479	69.9	71.5	61–82.5	60.6	55.4–69.6	−15.3
Hypertensive heart disease	36,179	30,771–46,101	62,663	55,680–71,029	73.2	27.0	22.7–35.2	21.4	19–24.1	−20.7
Cardiomyopathy and myocarditis	18,025	15,031–20,571	27,128	24,612–29,553	50.5	8.7	7.2–10	7.3	6.5–7.9	−16.6
Atrial fibrillation and flutter	3513	2654–4487	7535	5707–9666	114.5	3.9	2.9–5.1	3.5	2.6–4.5	−11.0
Aortic aneurysm	2694	2163–3414	6941	6291–7580	157.6	2.0	1.6–2.5	2.3	2.1–2.5	14.9
Peripheral artery disease	114	68–151	424	365–508	272.4	0.1	0.1–0.1	0.2	0.1–0.2	65.9
Endocarditis	5172	4167–7067	9016	7833–12,719	74.3	2.9	2.3–4.2	2.6	2.3–3.9	−8.3
Other cardiovascular and circulatory diseases	38,243	34,519–42,477	55,625	51,621–60,292	45.5	20.3	18.5–22.2	17.1	15.9–18.6	−15.7

The total number of deaths from ischemic heart disease (IHD) was 802,078 in 2015, which accounted for 58.4% of the total number of deaths due to CVD in the EMR. There were 637,640 additional deaths in 2015 compared to 1990, out of which 62.5% was contributed by IHD.

Table 2 provides the total number of deaths and the age-standardized death rates from CVD in 1990 and 2015 for all EMR countries. In 2015, Afghanistan had the highest age-standardized death rate from CVD, followed by Iraq and Yemen. In most of the EMR countries, age-standardized death rates for CVD decreased between 1990 and 2015, with the highest decreases in Bahrain, Qatar, Lebanon, and Jordan.

**Table 2:** Total number of deaths and age-standardized mortality rates for cardiovascular disease causes of death in 1990 and 2015, and percent change, Global Burden of Disease study, Eastern Mediterranean Region, 1990–2015

Country	Number of deaths					Age-standardized death rate per 100,000				
	1990		2015		% Change	1990		2015		% Change
	Number	95% UI	Number	95% UI		Rate	95% UI	Rate	95% UI	
EMR	735,689	700,875–773,593	1,373,329	1,290,959–1,465,047	86.7	515.1	491.7–541.5	456.5	431.5–484.2	−11.4
Afghanistan	34,755	27,217–42,776	10,1572	81,113–125,962	192.2	1048.1	860.6–1235.4	1042.5	865–1227.9	−0.5
Bahrain	614	547–681	792	671–933	29.0	414.1	371.4–456.9	186.1	162.1–210.2	−55.1
Djibouti	683	434–1025	1402	762–-2395	105.3	393.4	265.8–568.8	360.9	212.6–590.1	−8.3
Egypt	153,214	147,677–157,026	226,457	219,738–234,235	47.8	544.9	530.1–556.7	465.2	451.7–479.2	−14.6
Iran	96,775	86,347–107,587	176,299	148,576–203,480	82.2	499.2	451.4–547.5	402.2	344–456.9	−19.4
Iraq	44,476	38,326–51,342	75,604	61,673–91,552	70.0	657.6	569.1–755.1	604.4	503.7–715.3	−8.1
Jordan	4869	4319–5684	6788	6108–7611	39.4	416.0	370.2–481.4	236.9	214.1–264.4	−43.1
Kuwait	1262	1192–1324	2367	2040–2747	87.6	258.5	245–271.3	209.7	185–237	−18.9
Lebanon	7397	6206–8674	11,632	8967–14,195	57.3	464.2	391.3–540.9	252.1	196–305.1	−45.7
Libya	4864	4354–5397	9301	8130–10,535	91.2	310.3	276.6–344	299.7	263.3–339.3	−3.4
Morocco	36,293	32,487–40,581	59,824	47,641–75,972	64.8	362.1	327.5–400.4	268.3	216.5–336.6	−25.9
Oman	2108	1688–2552	4000	3336–4583	89.7	378.8	300.7–461.2	300.3	255.4–336.8	−20.7
Pakistan	216,936	191,002–247,476	465,116	407,279–528,666	114.4	513.1	454.9–578	530.9	469–599.1	3.5
Palestine	2333	1902–2925	5805	4683–6954	148.8	443.1	366.5–542	394.9	326.3–462.4	−10.9
Qatar	338	297–383	723	568–924	114.3	342.4	303.1–380.4	180.6	149.6–221.8	−47.3
Saudi Arabia	13,222	11,931–14,651	25,845	23,532–28,503	95.5	288.0	260.4–317.9	231.6	213.2–-253.4	−19.6
Somalia	11,706	3957–22,825	15,080	5270–31,505	28.8	508.9	192.7–890.2	439.7	172.6–813.3	−13.6
Sudan	42,922	35,852–51,825	74,648	56,697–97,015	73.9	611.3	512.7–738.5	501.9	388.7–634.1	−17.9
Syria	23,049	20,307–26,719	33,044	28,488–36,934	43.4	554.8	494.8–634.9	401.0	348.7–446.9	−27.7
Tunisia	10,747	9970–11,633	18,423	14,973–21,952	71.4	285.3	263.3–308.9	204.0	166.5–242.6	−28.5
UAE	1641	1260–2230	8563	6337–11,314	421.9	406.5	327.3–501.8	333.4	279.6–403.7	−18.0
Yemen	25,485	16,534–36,647	50,043	30,637–78,838	96.4	700.0	461.6–991.9	592.1	383–888.5	−15.4

Electronic supplementary figure S1 shows the top-ranked death rates for different CVD in EMR countries. Ischemic heart disease was the leading cause of CVD mortality in 20 countries of the EMR; the exceptions were Djibouti and Somalia, where cerebrovascular disease (both hemorrhagic and ischemic stroke) was the leading cause of cardiovascular-related death.

### YLLs

The age-standardized YLL rate decreased 15.3%, from 9618.7 (9148.6–10,141.7) per 100,000 in 1990–8145.0 (7628.6–8744.3) per 100,000 in 2015 (Electronic supplementary table S2). In the region, Afghanistan had the highest age-standardized YLL rate at 21,426.2 (17,105.2–26,544.7), followed by Yemen and Iraq (Electronic supplementary table S2). In all countries of the EMR except Pakistan, age-standardized YLL rates decreased from 1990 to 2015 (Electronic supplementary table S2).

### YLDs

The years lived with disability caused by CVD in the EMR increased from 1,058,839 (95% UI: 746,613–1409,913) in 1990 to 1,966,111 (95% UI: 1398,373–2597,819) in 2015. The rate of YLD increased by 85.7% during 1990–2015 in the EMR.

The age-standardized YLD rate in the EMR was 460.6 (329.2–603.6) per 100,000 in 2015, which showed very little decrease compared to 1990 (461.1 per 100,000) (Electronic supplementary table S2). Oman had the highest age-standardized YLD rate in the region in both 1990 and 2015: it was 1261 (874.6–1722.1) per 100,000 in 2015, which was about 2.7 times higher than the regional average. United Arab Emirates had the lowest age-standardized YLD rate in the EMR, 296.8 per 100,000 in 1990 and 285.8 per 100,000 in 2015. Age-standardized YLD rates of CVD decreased between 1990 and 2015 in six countries of the region: Iran, United Arab Emirates, Jordan, Djibouti, Somalia, and Afghanistan. The biggest decline was seen in Iran (4.6%), and the smallest reduction was in Afghanistan (0.5%). Among the remaining 16 countries of the region that showed increases in age-standardized YLD rates of CVD, Syria’s was the greatest, at 9.1%.

### DALYs

The rate of DALYs from CVD per 100,000 population decreased from 5447.8 (95% UI: 5168.2–5739.0) in 1990–5109.8 (95% UI: 4771.3–5511.1) in 2015, a 6.2% decrease—compared to an 8.4% reduction in the DALY rate for all other non-communicable diseases in the EMR. The age-standardized DALY rate also decreased 14.6% during 1990–2015 (Table 3). Table 3 reports numbers and age-standardized rates of DALYs for different CVD in the EMR in 1990 and 2015. The age-standardized DALY rate of CVD for men and women in the EMR in 2015 was higher than in other WHO regions. It was 1.51 times the global rate for males and 1.86 times the global rate for females. Electronic supplementary figure S2 shows the age-standardized rates of DALYs for different CVD in men and women. As shown, ischemic heart disease caused the highest number of DALYs both in men (5771.9 per 100,000) and women (3931.2 per 100,000), followed by hemorrhagic stroke and ischemic stroke.

**Table 3:** Total disability-adjusted life years (DALY) and age-standardized disability-adjusted life years rates for component cardiovascular causes of death in 1990 and 2015, and percent change, Global Burden of Disease study, Eastern Mediterranean Region, 1990–2015

Cause	Number of DALYs					Age-standardized DALY rate per 100,000				
	1990		2015		% Change	1990		2015		% Change
	Number	95% UI	Number	95% UI		Rate	95% UI	Rate	95% UI	
Cardiovascular diseases	20,164,206	19,129,504–21,242,151	33,131,948	30,937,166–35,734,353	64.3	10,079.8	9594.7–10,603.6	8605.6	8074.6–9219.3	−14.6
Rheumatic heart disease	876,838	770,813–986,361	1153,351	993,217–1333,219	31.5	302.1	265.1–344.5	215.8	185.2–248.4	−28.5
Ischemic heart disease	9323,188	8770,306–9877,741	17,827,201	16,511,324–19,368,534	91.2	5370.1	5052.6–5672.7	4865.0	4533.1–5231.4	−9.4
Ischemic stroke	1879,679	1649,862–2128,711	3272,789	2963,211–3568,718	74.1	1183.5	1031.4–1361	997.6	903.8–1085.4	−15.7
Hemorrhagic stroke	3941,327	3658,523–4323,670	5565,221	5091,084–6337,446	41.2	1649.0	1485.2–1870.5	1303.2	1193.9–1493	−21.0
Hypertensive heart disease	822,728	711,712–1011,157	1366,662	1201,763–1571,258	66.1	479.4	411.3–603	371.0	328.2–422.5	−22.6
Cardiomyopathy and myocarditis	833,292	693,153–982,453	1001,334	891,780–1097,777	20.2	247.2	206.1–281.7	188.0	170.3–204.4	−24.0
Atrial fibrillation and flutter	77,777	61,867–94,926	161,328	129,867–199,493	107.4	63.3	51.4–77	58.2	47–71	−8.0
Aortic aneurysm	63,221	50,928–80,451	163,304	146,635–180,105	158.3	35.4	28.5–45	41.6	37.6–45.6	17.6
Peripheral artery disease	13,954	7359–24,367	32,852	18,777–56,064	135.4	10.1	5.3–17.9	11.2	6.4–19.3	11.3
Endocarditis	217,347	160,723–291,718	292,842	248,013–378,079	34.7	68.4	55–92.5	58.5	50.9–80.3	−14.4
Other CVD	2114,855	1844,679–2463,667	2295,064	2018,478–2613,017	8.5	671.3	599.3–755.3	495.4	440.4–555.8	−26.2

Electronic supplementary figure S3 shows DALY rates for each CVD in different age groups. As shown, the highest DALY rates for IHD, hemorrhagic stroke, ischemic stroke, and hypertensive heart disease were observed in people aged 50–69 years. IHD, hemorrhagic stroke, and rheumatic heart disease showed the highest number of DALYs in the 15–49 years age group.

Table 4 summarizes age-standardized DALY rates for CVD in the EMR countries in 1990 and 2015. As shown, DALY rates decreased in all EMR countries except Pakistan from 1990 to 2015; the greatest reductions in DALY rates were seen in Bahrain (59.4%), Qatar (48.7%), and Jordan (47%). Afghanistan had the highest age-standardized CVD DALY rate in both 1990 and 2015. Kuwait had the lowest age-standardized DALY rate of CVD in 1990, and Qatar had the lowest in 2015.

**Table 4:** Total number of disability-adjusted life years and age-standardized disability-adjusted life years rates for cardiovascular diseases in 1990 and 2015, and percent change, 1990–2015, in Eastern Mediterranean Region countries

Country	Number of DALYs					Age-standardized DALY rate per 100,000				
	1990		2015		% Change	1990		2015		% Change
	Number	95% UI	Number	95% UI		Rate	95% UI	Rate	95% UI	
EMR	20,164,206	19,129,504–21,242,151	33,131,948	30,937,166–35,734,353	64.3	10,079.8	9594.7–10,603.6	8605.6	8074.6–9219.3	−14.6
Afghanistan	1,019,023	7,93,590–1,278,676	2,865,062	2,239,466–3,635,800	181.2	22,258.2	17,615.6–27,186.7	21,864.3	17,591.4–27,024.5	−1.8
Bahrain	18,578	16,524–20,639	23,376	19,748–27,808	25.8	8086.5	7206.4–8964	3281.4	2832.6–3769.2	−59.4
Djibouti	19,979	13,027–-29,586	35,930	19,430–64,153	79.8	7891.5	5171.4–11,605.5	7112.8	4021.2–12,056.6	−9.9
Egypt	4,373,017	4,109,349–4,595,897	5,436,416	5,216,937–5,700,475	24.3	11,230.6	10,762.9–11,583.1	8826.2	8508.6–9171.3	−21.4
Iran	2,941,466	2,601,284–3,291,417	3,875,985	3,249,465-4,577,119	31.8	9849.9	8776.8–10,950.2	7179.6	6090.9–8340.9	−27.1
Iraq	1,070,614	917,957–1248,583	1,875,448	1,489,313–2,315,419	75.2	12,513.4	10,678.3–14,669.9	11,244.0	9089.6–13,679.3	−10.1
Jordan	109,195	95,903–127,032	154,251	137,970–172,312	41.3	7692.8	6771.4–8983.8	4077.5	3666.1–4533	−47.0
Kuwait	40,918	38,315–43,123	75,385	65,509–87,507	84.2	4818.8	4558.7–5066.9	3884.1	3424.2–4417.1	−19.4
Lebanon	167,913	140,761–199,069	211,244	159,897–264,585	25.8	8792.7	7390.6–10,364.6	4213.8	3210.3–5249.2	−52.1
Libya	176,223	154,977–198,183	234,502	205,521–265,135	33.1	6384.4	5714.7–7050.6	5638.4	4957.2–6367.8	−11.7
Morocco	1,103,861	980,405–1,235,540	1,332,750	1,078,637–1,670,227	20.7	7222.4	6511.3–8059.2	4977.5	4039.5–6209.9	−31.1
Oman	93,965	76,349–115,695	135,300	114,087–155,337	44.0	8404.3	6832–10,016.2	5962.4	5071–6713.2	−29.1
Pakistan	5,069,303	4,422,395–5,880,731	10,719,663	9,250,078–12,360,492	111.5	9446.3	8281–10,798.4	9928.0	8664.3–11,288.5	5.1
Palestine	68,438	55,953–85,314	150,510	120,084–183,652	119.9	8263.4	6749.8–10,377.3	7280.6	5868.1–8727.7	−11.9
Qatar	10,556	9283–12,017	24,791	19,932–30,601	134.8	5873.6	5192.9–6542.3	3013.6	2466.4–3730	−48.7
Saudi Arabia	359,601	320,837–401,588	663,879	600,438–732,764	84.6	5285.0	4727.5–5861.6	4003.3	3650.2–4393.1	−24.3
Somalia	329,146	120,719–676,354	410,106	151,542–920,717	24.6	10,762.8	3851–21,078.4	9062.4	3379.7–18,999.5	−15.8
Sudan	1,359,599	1,129,865–1,596,275	2,047,475	1,542,545–2,657,659	50.6	12,814.4	10,687.4–15,522.9	9823.7	7427.1–12,742.4	−23.3
Syria	668,927	579,151–779,665	766,383	663,438–864,601	14.6	11,211.5	9876.4–13,008.3	7277.4	6299.5–8180.1	−35.1
Tunisia	282,490	259,822–306,247	371,042	306,673–438,530	31.3	5367.9	4992.8–5778.7	3694.6	3055.8–4362	−31.2
UAE	56,629	42,431–80,907	304,764	220,613–401,976	438.2	7978.3	6300–10,281.2	6184.6	4945.5–7774.5	−22.5
Yemen	824,766	550,243–1,143,803	1,417,685	876,250–2,252,582	71.9	14,715.3	9437–21,422.1	11,692.8	7228.8–18,372.2	−20.5

Analyzing the components of DALYs, CVD had a higher YLL rate compared to YLD rate: on average, YLLs were 17.7 times higher than YLDs in the EMR. The YLL/YLD ratio in the countries of the region showed a wide range of variation, from 48.9 in Afghanistan to 3.7 in Oman (Electronic supplementary table S2).

### Risk factors

**Fig. 2:**
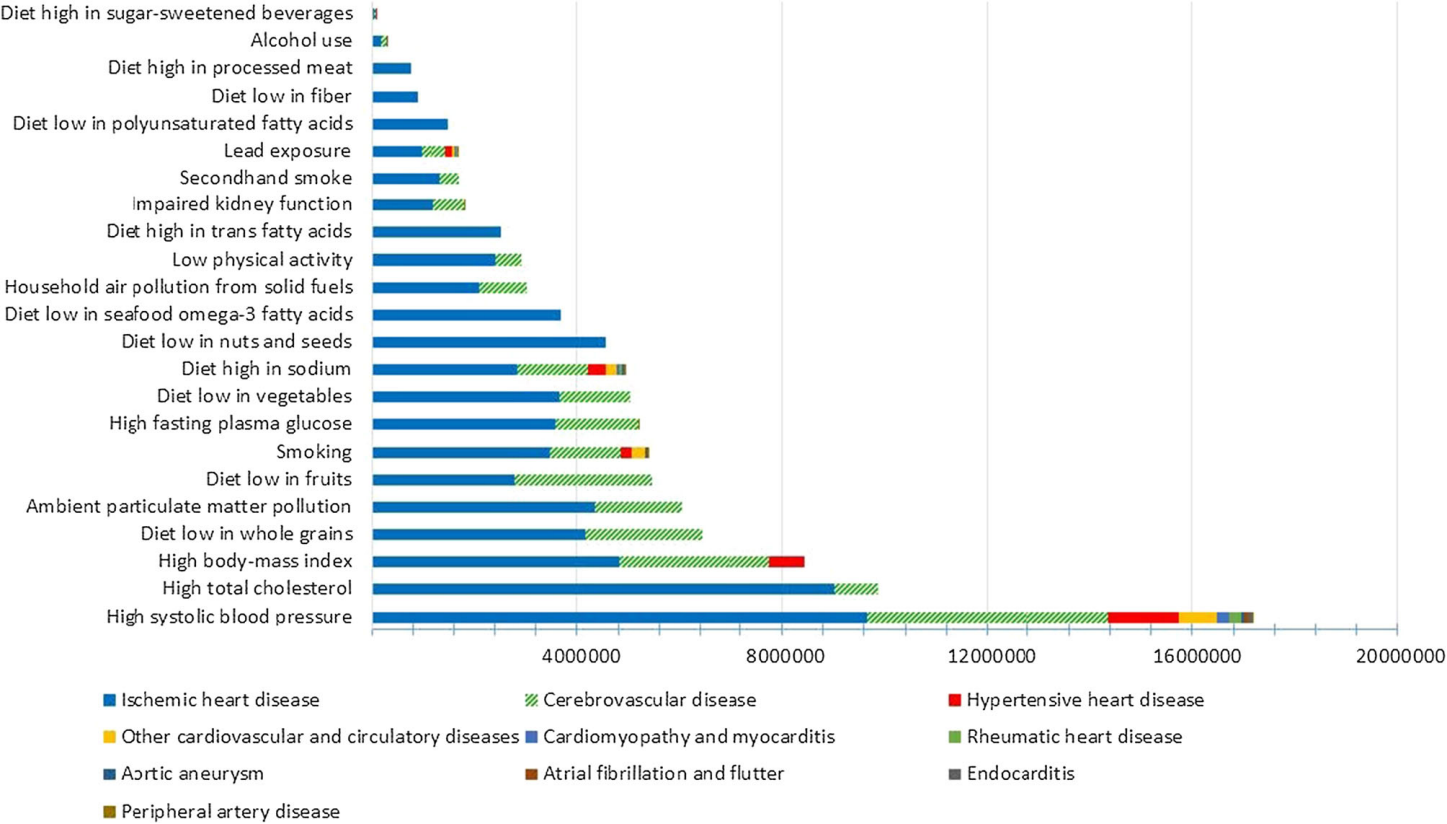
Number of disability-adjusted life years for different cardiovascular diseases attributed to different risk factors, Global Burden of Disease study, Eastern Mediterranean Region, 2015

Figure 2 shows the contribution, in DALYs, of different risk factors to different CVD. High blood pressure, high total cholesterol, and high body mass index were the leading risk factors for CVD, accounting for 17,159,331 DALYs, 9852,820 DALYs, and 8427,021 DALYs, respectively.

The cluster of all dietary risk factors accounts for 19,803,725 DALYs, making it the leading risk factor for CVD, higher than even high blood pressure. Low whole grains, low fruit, low vegetables, and high sodium intake were the most important dietary risk factors.

## Discussion

This study shows that CVD are the leading cause of disease burden in the EMR as a whole and in most of the countries of the region. Close to 33 million years of life were lost due to premature mortality or disability from CVD, and more than 1.3 million people died in the EMR in 2015, accounting for around one-third of all deaths in the region. Previous studies have also reported CVD deaths as the main cause of death, for instance, 45% in the West Bank (Palestine), 45% in Aleppo (Syria), 35% in Jordan, and 25% in UAE (Barakat et al. 2012; Loney et al. 2013; Shara 2010). A study in Europe has reported CVD mortality as making up half of all deaths (Nichols et al. 2014).

CVD age-standardized mortality was considerably higher than the global average (456 compared to 286 per 100,000); however it shows a declining trend over the past 25 years in most of the EMR countries. Countries with higher declines (Bahrain, Qatar, Lebanon, and Jordan) were among the countries in the fourth Socio-demographic Index quartile category. In another GBD study, we estimated an index for healthcare access and quality which is a composite index based on estimates of mortality amenable to personal health care and varies between 0 (worst) and 100 (best). The index showed substantial heterogeneity with a range between 32 (Afghanistan) and 85 (Qatar) in 2015 in the EMR. Linking these results to the findings of our study showed that the countries with lower age-standardized DALY rates due to CVD had a higher index for healthcare access and quality, and vice versa. This restates the importance of increasing access to and quality of health care to reduce CVD burden (Barber et al. 2017).

In the EMR, YLLs are the main component of CVD burden. A global-level assessment showed that for overall CVD, YLL rates were lowest in both the lowest and highest socio-demographic groups, with an increase for those in the middle of the socio-demographic rankings. It has been suggested that medical care in countries with the highest Socio-demographic Index might have increased life expectancy to the point where CVD is most prevalent, while people in the lowest socio-demographic group are dying from other competing conditions before reaching the common age for developing ischemic heart disease and stroke. Based on this hypothesis, people living in countries in the middle range of the socio-demographic rankings are surviving long enough to develop ischemic heart disease but do not have access to optimal medical or surgical treatment (GBD 2015 Mortality and Causes of Death Collaborators 2016).

These findings call for a comprehensive approach to prevent and control the burden of CVD in the region. This approach should include a road map for better monitoring of the burden in EMR countries, with a focus on potential variations in risk and care by regions within the countries. It should also include programs for increasing awareness among the general population of the importance of controlling CVD risk factors.

The United Nations has set targets to decrease mortality from non-communicable diseases (Sustainable Development Goals, target 3.4.1), and CVD is at the center of this target (GBD 2015 SDGs Collaborators 2016). The World Health Organization has suggested a package of essential non-communicable disease interventions for primary health care in low-resource settings (PEN). These interventions include a mixture of cost-effective population-wide and individual approaches to reduce the burden of major non-communicable diseases, such as methods for early detection and diagnosis using inexpensive technologies, non-pharmacological and pharmacological approaches for modification of risk factors, and affordable medications for prevention and treatment of heart attacks and strokes, diabetes, cancer, and asthma (World-Health-Organization 2010).

Our study showed that increased blood pressure is the most important risk factor for CVD in the EMR, followed by high total cholesterol and high body mass index. A Cochrane systematic review showed that multiple risk factor interventions may lower systolic and diastolic blood pressure, body mass index, and waist circumference in low- and middle-income countries (Uthman et al. 2015).

Previous studies show a high percentage of undiagnosed CVD risk factors, such as diabetes and hypertension, in the region (Abd El-Aty et al. 2015; El Bcheraoui et al. 2014a, b; Najafipour et al. 2014). The evidence shows that delayed detection and undiagnosed risk factors, especially diabetes, are strong predictors of fatal CVDs (Nakagami et al. 2006). Based on reports from the region, required care and services (such as medications) are underutilized in diagnosed cases, even in high-income countries like Saudi Arabia (Moradi-Lakeh et al. 2016). Underutilization of medications is a function of availability, accessibility, affordability, acceptability, and quality of medicines (and care), as well as adherence to medical recommendations (Behnood-Rod et al. 2016; Najafipour et al. 2014; van Mourik et al. 2010; Wirtz et al. 2016). The Prospective Urban Rural Epidemiology (PURE) study showed great variation in availability, affordability, and use of medications for CVD, between and within countries. Countries with less control over production, importation, distribution chains, and retail outlets are specifically at risk of substandard quality and falsification of medicines (Khatib et al. 2016). All these factors are important to achieve desired health outcomes in the field of CVD. CVD prevention and control programs should improve the perceived need and demand of the population for early detection and use of the prevention/control services. The study on CVD mortality forecast in 2015 has shown that the MENA region will not achieve the target of 25% reduction of CVD mortality by 2025 without achieving all major targets for risk factor reduction (i.e., reducing the prevalence of elevated systolic blood pressure by 25%, reducing the prevalence of smoking by 30%, halting the rise in elevated body mass index, and halting the rise in fasting plasma glucose). Moreover, reports of health system challenges in controlling and managing CVD in some of the EMR countries reemphasize the need for significant investment and improvement of access (Roth et al. 2015a, b; Romdhane et al. 2015; Ahmad et al. 2015).

Our study has some limitations; accurate data on cardiovascular events (especially non-fatal outcomes) are limited in many countries, including the EMR countries. We used the standard GBD methodology by using study- and country-level covariates for adjustment and estimation of epidemiologic measures. Our study does not account for variation within countries.

### Conclusion

Most of the EMR countries have launched programs to reduce the burden of non-communicable disease, but they generally do not have widespread programs to combat CVD. This study calls for strengthening efforts to design and launch comprehensive programs to cover all aspects of prevention and control of CVDs through evidence-informed, efficient interventions. The countries should establish or improve information systems such as surveillance sy stems to provide valid and accurate information for policymaking and monitoring of the situation.

## Electronic supplementary material

Below is the link to the electronic supplementary material.

Supplementary material 1 (XLSX 26 kb)

Supplementary material 2 (DOCX 259 kb)
