# Supplementary material for: Burden of cardiovascular diseases in the Eastern Mediterranean Region, 1990–2015: findings from the Global Burden of Disease 2015 study
Source: Int J Public Health. 2017 Aug 3;63(Suppl 1):137–49. doi: 10.1007/s00038-017-1012-3 (PMC5973984; doi:10.1007/s00038-017-1012-3)
Supplement: Supplementary file 2 — Supplementary material 2 (DOCX 259 kb) [file 38_2017_1012_MOESM2_ESM.docx]

Electronic Supplementary Material

**Article title:**

Burden of cardiovascular diseases in the Eastern Mediterranean Region, 1990–2015: Findings from the Global Burden of Disease 2015 study

**Journal:**

International Journal of Public Health

**Authors:**

GBD 2015 Eastern Mediterranean Region Cardiovascular Disease Collaborators

**Corresponding author:**

Ali H. Mokdad

Institute for Health Metrics and Evaluation, University of Washington, Seattle, WA, United States

Email: [mokdaa@uw.edu](mailto:mokdaa@uw.edu)


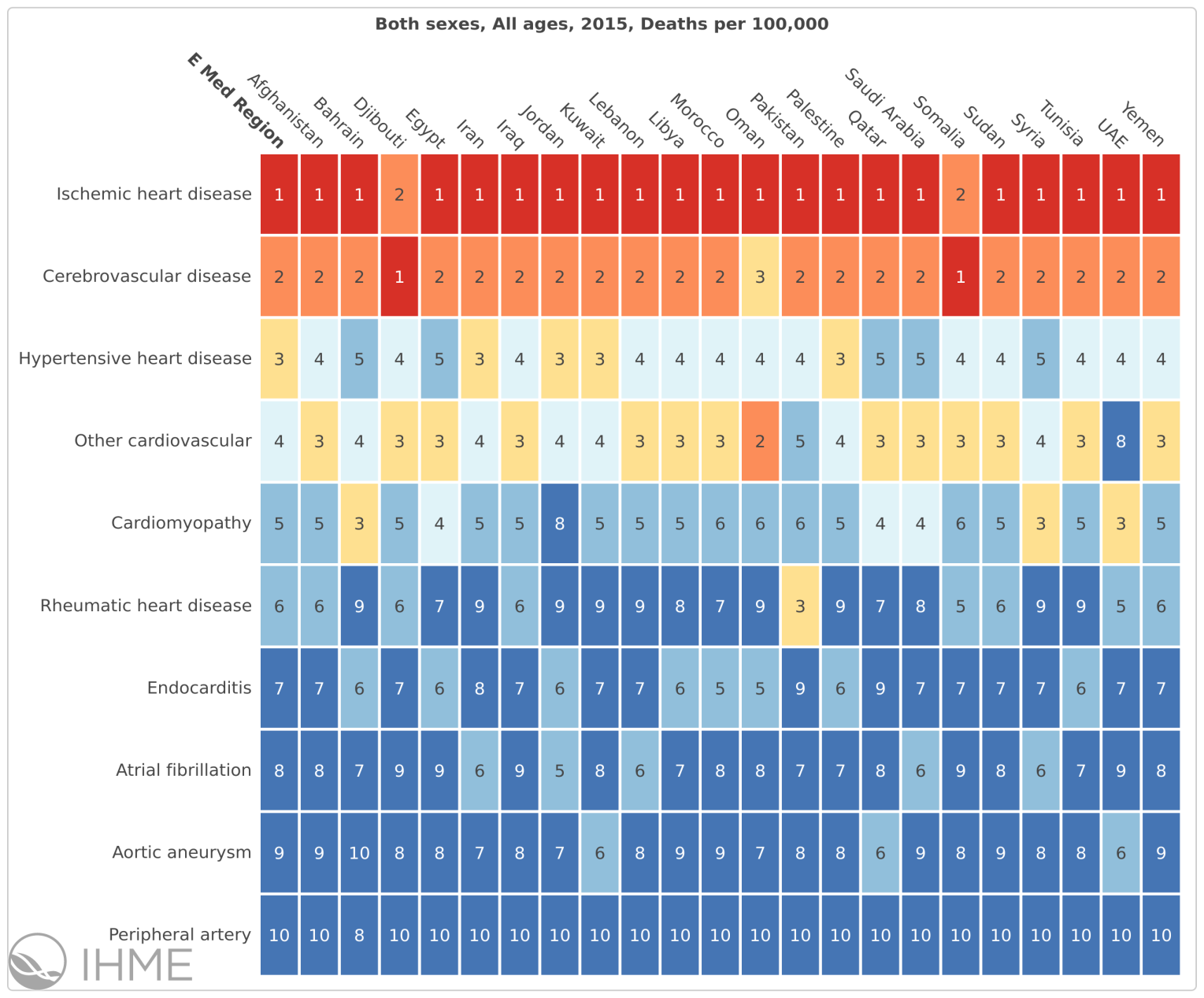


Electronic supplementary figure S1. Top-ranked cardiovascular diseases, by death rates, Global Burden of Disease study, Eastern Mediterranean Region, 2015

Electronic supplementary figure S2. Age-standardized rates of disability-adjusted life years for different cardiovascular diseases in the Global Burden of Disease study, Eastern Mediterranean Region, 2015

Electronic supplementary figure S3. Number of disability-adjusted life years for different cardiovascular diseases, Global Burden of Disease study, Eastern Mediterranean Region, 2015

**Electronic supplementary table S1. International Classification of Disease (ICD) 10 codes used for modeling of cardiovascular diseases, Global Burden of Disease, Eastern Mediterranean Region, 1990–2015**

| **Cause** | **Cause of death ICD-10 codes** | **Morbidity cause ICD-10 codes** |
| --- | --- | --- |
| Rheumatic heart disease | I01-I01.9, I02.0, I05-I09.9 | I01-I01.2, I01.8-I01.9, I02.0, I05-I05.2, I05.8-I05.9, I06-I06.2, I06.8-I06.9, I07-I07.2, I07.8-I07.9, I08-I08.3, I08.8-I08.9, I09-I09.2, I09.8-I09.9 |
| Ischemic heart disease | I20-I25.9 | I21-I21.4, I21.9, I22-I22.2, I22.8-I22.9 |
| Cerebrovascular disease | G45-G46.8, I60-I61.9, I62.0-I62.03, I63-I63.9, I65-I66.9, I67.0-I67.3, I67.5-I67.6, I68.1-I68.2, I69.0-I69.398 | I68-I68.2, I69-I69, I69-I69.3  I60-I60.9, I61-I61.6, I61.8-I61.9, I62.0, I63-I63.6, I63.8-I63.9, I67-I67.7 |
| Hypertensive heart disease | I11-I11.9 |  |
| Cardiomyopathy and myocarditis | A39.52, B33.2-B33.24, D86.85, I40-I43.9, I51.4-I51.5 | A39.5, B33.2, I40-I40.1, I40.8-I40.9, I41-I41.2, I41.8-I41.9, I51.4-I51.6 |
| Atrial fibrillation and flutter | I48-I48.92 | I48-I48.4, I48.9 |
| Aortic aneurysm | I71-I71.9 | - |
| Peripheral vascular disease | I70.2-I70.799, I73-I73.9 | I70-I70.9, I73-I73.1, I73.8-I73.9 |
| Endocarditis | A39.51, I33-I33.9, I38-I39.9 | A32.8, A39.5, A52.0, B33.2, B37.6, I33.0, I33.9, I38.0, I38.9, I39-I39.4, I39.8-I39.9, M32.1 |
| Other cardiovascular and circulatory diseases | A39.5-A39.50, A39.53, I28-I28.8, I30-I31.1, I31.8-I32.8, I34-I37.9, I47-I47.9, I51.0-I51.3, I68.0, I72-I72.9, I77-I83.93, I86-I89.9, I91.9, I98 | - |

| **Electronic supplementary table S2. Age-standardized years of life lost (YLL) and years lived with disability (YLD) rates and YLL/YLD ratios for cardiovascular diseases in 1990 and 2015, Global Burden of Disease study, Eastern Mediterranean Region, 1990–2015** | | | | | | | | | | |
| --- | --- | --- | --- | --- | --- | --- | --- | --- | --- | --- |
| **country** | **Age-standardized YLD rate per 100,000** | | | | **Age-standardized YLL rate per 100,000** | | | | **YLL/YLD ratio  1990** | **YLL/YLD ratio  2015** |
|  | **1990** | | **2015** | | **1990** | | **2015** | |  |  |
|  | **rate** | **95% UI** | **rate** | **95% UI** | **rate** | **95% UI** | **rate** | **95% UI** |  |  |
| EMR | 461.1 | 329.8–605.4 | 460.6 | 329.2–603.6 | 9,618.7 | 9,148.6–10,141.7 | 8,145.0 | 7,628.6–8,744.3 | 20.9 | 17.7 |
| Afghanistan | 440.3 | 318.6–574.6 | 438.1 | 314.5–570.5 | 21,817.9 | 17,140.4–26,735.3 | 21,426.2 | 17,105.2–26,544.7 | 49.6 | 48.9 |
| Bahrain | 401.4 | 284.4–524.5 | 418.2 | 297.7–552.8 | 7,685.1 | 6,831–8,548 | 2,863.3 | 2,430.6–3,346.3 | 19.1 | 6.8 |
| Djibouti | 559.2 | 395.5–757.5 | 554.6 | 395.5–746 | 7,332.3 | 4,617.7–11,087 | 6,558.2 | 3,448.4–11,506.3 | 13.1 | 11.8 |
| Egypt | 428.7 | 305.1–565.1 | 448.0 | 321.4–586.4 | 10,801.9 | 10,366.2–11,107.8 | 8,378.2 | 8,110.4–8,728.3 | 25.2 | 18.7 |
| Iran | 721.0 | 503.4–959.2 | 688.1 | 483.3–924.9 | 9,129.0 | 8,090.8–10,182.6 | 6,491.5 | 5,394.7–7,642.1 | 12.7 | 9.4 |
| Iraq | 456.9 | 326.6–598 | 466.9 | 334.8–608.2 | 12,056.5 | 10,242.9–14,137.6 | 10,777.1 | 8,670.2–13,228.3 | 26.4 | 23.1 |
| Jordan | 403.2 | 285.6–530.2 | 399.1 | 283.8–526.1 | 7,289.6 | 6,388.7–8,602.4 | 3,678.5 | 3,298.9–4,139.9 | 18.1 | 9.2 |
| Kuwait | 374.1 | 266.1–492.4 | 394.9 | 280–520.2 | 4,444.7 | 4,205.2–4,660.4 | 3,489.3 | 3,042.6–4,006.4 | 11.9 | 8.8 |
| Lebanon | 372.1 | 267.1–490.1 | 378.8 | 270.6–502 | 8,420.6 | 7,012.7–10,036.1 | 3,835.0 | 2,836–4,865.4 | 22.6 | 10.1 |
| Libya | 416.6 | 296.4–549.4 | 443.5 | 313.4–585.2 | 5,967.8 | 5,327.7–6,623.2 | 5,194.9 | 4,544.6-5,887.9 | 14.3 | 11.7 |
| Morocco | 552.2 | 391.5–732.2 | 567.4 | 401.3-754 | 6,670.2 | 5,955.4–7,493.9 | 4,410.1 | 3,460.8–5,669.6 | 12.1 | 7.8 |
| Oman | 1,229.4 | 855.2–1,673.3 | 1,261.0 | 874.6–1,722.1 | 7,174.9 | 5,614.9–8,765.2 | 4,701.4 | 3,900.4–5,380.3 | 5.8 | 3.7 |
| Pakistan | 348.1 | 252–453.7 | 355.3 | 254.9–463.4 | 9,098.2 | 7,952.4–10,431.4 | 9,572.7 | 8,303–10,939.4 | 26.1 | 26.9 |
| Palestine | 347.6 | 246.6–454.8 | 364.1 | 256.4–477.9 | 7,915.8 | 6,403.1–10,027.4 | 6,916.5 | 5,530.1–8,331.7 | 22.8 | 19.0 |
| Qatar | 409.5 | 289–543.4 | 421.8 | 298.7–558.1 | 5,464.0 | 4,828.8–6,151.1 | 2,591.8 | 2,074.5–3,270.7 | 13.3 | 6.1 |
| Saudi Arabia | 425.8 | 305.5–561.9 | 448.1 | 320.1–593.4 | 4,859.2 | 4,353.2–5,398.8 | 3,555.2 | 3,225.5–3,927.2 | 11.4 | 7.9 |
| Somalia | 522.8 | 373.6–702.8 | 518.8 | 376–694.3 | 10,240.0 | 3,323–20,520.4 | 8,543.6 | 2,851.4–18,507.3 | 19.6 | 16.5 |
| Sudan | 465.9 | 331–610.7 | 483.8 | 344.6–636.8 | 12,348.5 | 10,217.2–15,010 | 9,339.8 | 7,003.3–12,265.6 | 26.5 | 19.3 |
| Syria | 332.2 | 239.9–435.2 | 362.5 | 259.3–476.4 | 10,879.3 | 9,542.3–12,690.9 | 6,914.9 | 5,931.1–7,816.3 | 32.7 | 19.1 |
| Tunisia | 480.8 | 342.4–635.2 | 502.8 | 356.8–664 | 4,887.2 | 4,531.2–5,289.7 | 3191.8 | 2,585.5–3,844.4 | 10.2 | 6.3 |
| UAE | 296.8 | 215.2–385.9 | 285.8 | 206.5–374 | 7,681.5 | 5,980.5–9,984.8 | 5898.8 | 4,674.6–7,504.8 | 25.9 | 20.6 |
| Yemen | 444.1 | 319.4–583.2 | 451.0 | 323.8–591.8 | 14,271.2 | 8,961.1–20,938.8 | 11241.8 | 6,821.5–17,849.8 | 32.1 | 24.9 |
